# Case Report: Sellar Ependymomas: A Clinic-Pathological Study and Literature Review

**DOI:** 10.3389/fendo.2021.551493

**Published:** 2021-06-08

**Authors:** Liyan Zhao, Yining Jiang, Yubo Wang, Yang Bai, Liping Liu, Yunqian Li

**Affiliations:** ^1^Department of Clinical Laboratory, Second Hospital of Jilin University, Changchun, China; ^2^Department of Neurosurgery, First Hospital of Jilin University, Changchun, China

**Keywords:** diagnosis, ependymoma, molecular subtype, pituitary tumor, sellar ependymoma, treatment

## Abstract

Ependymomas are primary glial tumors arising from cells related to the ependymal lining of the ventricular system. They are classified into at least nine different molecular subtypes according to molecular phenotype, histological morphology, and tumor location. Primary sellar ependymoma is an extremely rare malignancy of the central nervous system, with only 12 known cases reported in humans. We herein report a case of ependymoma located at the pituitary region in a 44-year-old female patient and discuss the molecular subtype, natural history, clinical presentation, radiological findings, histological features, immunohistochemical characteristics, ultrastructural examinations, treatment, and prognosis of sellar ependymoma. This case report may serve as a helpful reference for clinicians and radiologists in clinical practice.

## Introduction

Ependymomas are relatively uncommon primary glial tumors, accounting for 2% to 6% of the central nervous system (CNS) tumors (1), that usually arise from the ependymal lining of the cerebral ventricles, the central cord of the spinal canal, and/or terminal ventricle cells in the terminal filum (2–4). Ependymomas that develop in brain parenchyma are rare, and exceptionally rare in saddle areas (2, 3, 5). Upon reviewing all the primary ependymoma reports available on PubMed, only 12 cases were reported in humans (3, 5–15). Notably, all reported cases were diagnosed by pathological confirmation after surgical resection. Here, we report a case of sellar ependymoma (SE) involving a 44-year-old female with a history of 7 months of secondary amenorrhea and bitemporal hemianopsia. Magnetic resonance imaging (MRI) showed a mass located in the sellar and suprasellar region of the brain. The patient underwent total microsurgical removal and was found to have an ependymoma of Grade II classification. In addition, we reviewed the available medical and scientific literature and summarized all the reported SE cases, as well as the etiology, radiological features, histopathological characteristics, differential diagnoses, current treatment landscape, and prognosis of this rare tumor entity.

## Case Report

### History and Examination

A 44-year-old female was admitted to the Neurosurgery Department with a 7-month history of intermittent headaches, secondary amenorrhea, and visual blurring. The patient reported not having galactorrhea, hyponatremia, hyposexuality, weakness, or any other sensory or motor dysfunctions. A review of the family history indicated no history of tumor or cancer in close family members. Moreover, neuro-ophthalmologic examination disclosed bitemporal hemianopia, which was worse in the right eye, and no papilledema was found. A Snellen chart indicated a 0.4 value in the unaided right eye and 0.7 in the left eye. A preoperative laboratory examination showed a notable increase in the prolactin (PRL) level (1,370.51 mIU/L, range: 70.81-566) and no evidence of pituitary dysfunction or hypoadrenocorticism.

### Neuroimaging Findings

**Figure 1:**
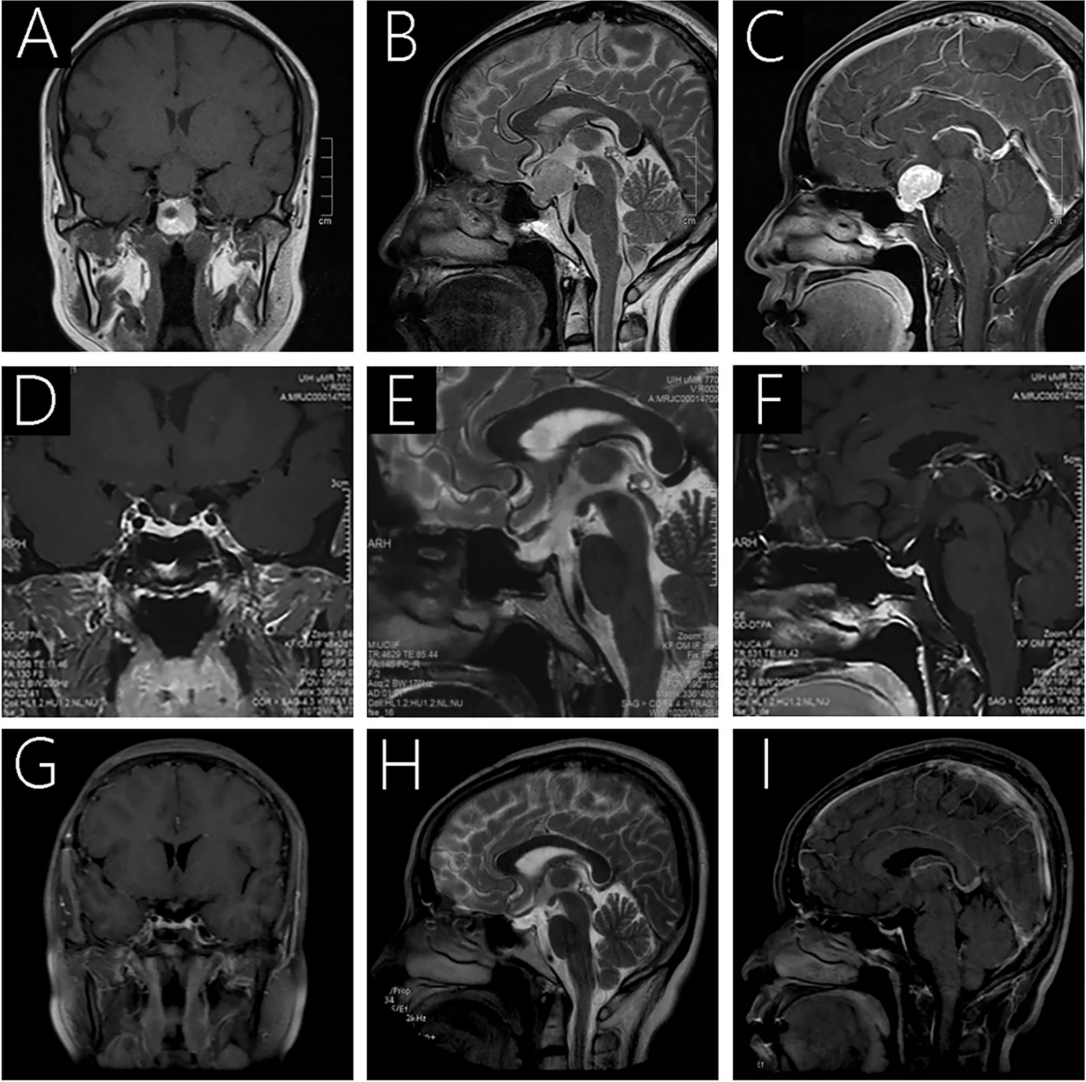
Preoperative MRI **(A-C)** revealed a 1.94×1.91×2.16-cm well-defined mass located in the sellar and suprasellar regions. It was isointense on T1WI **(A)** and slightly hyperintense on T2WI **(B)**, and the tumor was strongly and irregularly contrast-enhanced after gadolinium administration **(C)**. Follow-up MRI 3 months **(D–F)** and 27 months after surgery **(G–I)** showed complete removal of the lesion, with no sign of recurrence.

MRI scanning revealed a 1.94×1.91×2.16-cm (anterior-posterior×transverse×cranial-caudal) well-defined mass located in the sellar and suprasellar regions of the brain (Knosp II and Hardy-Wilson Grade III), with a noticeable expansion of the sella turcica. Furthermore, the tumor was isointense on the T1-weighted imaging (T1WI; **Figure 1A**), slightly hyperintense on the T2-weighted imaging (T2WI; **Figure 1B**), with strong and irregular contrast enhancement after gadolinium administration (**Figure 1C**). Notably, the tumor compressed the overlying optic chiasm, and there was no evidence of cavernous sinus invasion. In addition, the pituitary stalk was difficult to locate as the lesion protruded to the front of the brain third ventricle.

### Surgery

A preoperative clinical diagnosis of pituitary adenoma was made and a total surgical resection was performed by the corresponding author using a transfrontal-temporal approach. The tumor appeared pinkish in color, soft, friable, rich in vascular supply and was found located near the junction with the optic chiasm. The surgery was uneventful, with no noticeable leakage of cerebrospinal fluid or substantial venous bleeding. Follow-up MRI analysis demonstrated a total excision of the tumor (**Figures 1D–I**).

### Histopathology Findings

**Figure 2:**
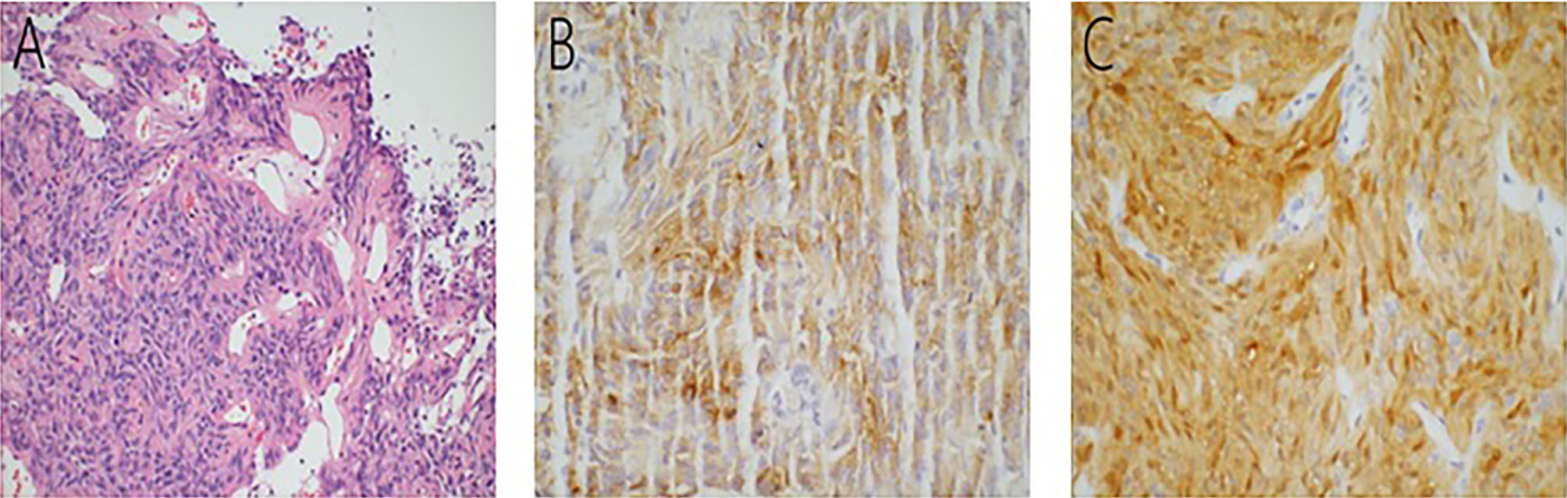
Well-formed perivascular anucleate zones (pseudorosettes) and (true) ependymal rosettes composed of bland cuboidal or columnar tumor cells seen throughout the tumor **(A)**. Immunolabeling was positive for EMA **(B)** and S-100 **(C)**.

The postoperative pathology manifested SE of World Health Organization (WHO) Grade II classification. Moreover, gross specimen sections showed a gray-brown, soft, and friable mass. When examining hematoxylin and eosin (H&E)-stained paraffin sections, we observed characteristic and well-formed perivascular anucleate zones (pseudorosettes) and (true) ependymal rosettes composed of bland cuboidal or columnar tumor cells (**Figure 2A**). Moreover, immunohistochemical analysis showed that the Ki-67 labeling index for cell division was approximately 3%. Positive staining was found for thyroid transcription factor-1 (TTF-1), neuron-specific enolase, vimentin, and epithelial membrane antigen (EMA; **Figure 2B**), as well as S-100 (**Figure 2C**). In addition, our staining results were negative for glial fibrillary acidic protein (GFAP), synaptophysin, and pituitary hormones. Unfortunately, no further genetic tests were performed.

### Postoperative Course

Visual blurring significantly improved after surgery, and the patient was discharged without any neurological deficits on day 14. A postoperative laboratory examination revealed adrenocortical axis and thyroid axis hypofunction, and the patient underwent disciplinary hormone replacement therapy for six months. Follow-up MRI scans were taken three months postoperatively (**Figures 1D–F**), demonstrating that the lesion had been completely removed, with no sign of recurrence. During the last follow-up in April 2021 (27 months postoperatively), the patient reported leading a normal daily life without noticeable symptoms, and the follow-up MRI exhibited no recurrence (**Figure 1G-I**). We believe that her condition is stable and will continue to monitor her progress with routine follow-up MRIs.

## Discussion

Ependymomas are primary glial tumors that arise from cells related to the ependymal lining of the cerebrospinal fluid circulatory system (9, 16). The saddle area is an exceedingly rare location for ependymomas, first reported by Sarkisian and Schultz in 1956 (6). Since then, only 12 cases of primary SEs have been reported in humans (**Tables 1**, **2**). Interestingly, Heath et al. also reported a case of a pituitary ependymoma occurrence in a horse (17). In 2017 WHO classification of pituitary tumors, SE, pituicytoma, granular cell tumor, and spindle cell oncocytoma were classified as nosological entities, called primary posterior pituitary tumors, a distinct group of low-grade and non-neuroendocrine neoplasms with TTF-1 immunopositivity (18, 19). All these tumors are believed to arise from pituicytes (20). Among them, SE is the least described, and scant effective information is available (18–22). According to the latest histopathological classification of WHO in 2016, ependymoma was classified into three grades (23). Grade I: subependymoma and myxopapillary ependymoma; Grade II: papillary ependymoma, clear cell ependymoma, and tanycytic ependymoma; Grade II-III: ependymoma, RELA fusion-positive; Grade III: anaplastic ependymoma. For ependymomas of Grade I, the prognosis is usually good, and it can be cured by surgery alone (24). For ependymomas in Grades II and III, the differential diagnosis is difficult. Increased cell density, nuclear atypia, a high mitosis rate, and varying degrees of angiogenesis are the main features of ependymomas in Grade III for differentiation (25). However, in some clinical settings, some literature presented a poor association between tumor grading and outcome (25).

**Table 1:** Patients’ characteristics of reported case in the literature.

Author^reference^/year	Age(y)/sex	Main symptoms and signs	Laboratory Examination	CT	MRI	Initial Treatment	Radiotherapy	Recurrence	Follow-up (months)
Present case	44/F	Secondary amenorrhea for 7 months; loss of vision; bitemporal hemianopsia.	PRL: 1370.51 mIU/L (70.81-566); Serum COR 0am:66.45 nmol/L (240-619);	N/A	A 1.94×1.91×2.16cm well-defined mass locating in the sellar and suprasellar region. Iso- was seen on T1WI and slight hyper- on T2WI, and the tumor was significantly enhanced on enhancement. It compressed the overlying optic chiasm.	Craniotomy: GTR	N	N	Y, follow-up for 15 months with stable condition, without recurrence or residual tumor.
Liu (13)/2019	35/M	Visual blurring without obvious incentive for 16 days; sexual hypoactivity for 4 months.	N/A	The lesion was located in sellar region, presented iso- and hypodensity, nodular calcification can be observed around.	The lesion was was featured by plastic growth and involved the 3^rd^ ventricle superiorly, the interpeduncular cistern posteriorly, and prepontine cistern inferiorly. The lesion showed slightly hypo- T1 and slightly hyper- T2 signals, iso- on DWI, flow-void and evident heterogeneous enhancement on T1 enhanced.	Craniotomy: Much of the mass resection.	N/A	N/A	N/A
Wang (3)/2018	35/M	The right eye visual deterioration for more than 1 year.	Serum COR 4pm: 4.6mg/dL (8.7-22.6);	Without CT, but the X-Ray showed expanded sella turcica, eroded dorsum sellar, and thinned sellar floor. Sign of double sellar floor was obvious.	A sharply circumscribed lesion with slightly hypo T1 and slightly hyper T2. Significantly homogeneous enhancement on T1 enhanced. The lesion compressed the overlying optic chiasm, bilateral cavernous sinus and internal carotid artery. The 3rd ventricle was elevated and compressed.	Transsphenoidal approach: GTR	Y, three dimensionalconformal radiotherapy/4 fields/54 Gy/1.8Gy/ 30f for 5 months.	N	Y, follow-up for 36 months with stable condition, without recurrence.
Lee (12)/2017	59/M	Fatigue; general weakness; erectile dysfunction; loss of body hair; visual blurring and bitemporal hemianopsia.	Panhypopituitarism; FT4: 0.39ng/dL (0.89–1.79); serum COR: <1.0µg/dL (4.3–22.4); T<0.01ng/mL (6-60);	N/A	A 3.3×3.5×2.3cm snowman-shaped mass involved sellar and suprasellar region, and compressed the overlying optic chiasm; hypo-enhanced homogeneous solid mass.	Transsphenoidal approach: Partial resection.	Y, 54 Gy over 30 fractions to sellar lesion for 6 weeks	N	Y, 10 years follow-up without recurrence.
Parish (14)/2015	46/M	Fatigue; loss of libido; erectile dysfunction; mood swings; loss of body hair; slight bitemporal superior quadrantanopia.	Panhypopituitarism. Not given for detailed hormonal changes.	N/A	Solid-cystic mass originating and extending out of the enlarged sellar and elevating the optic chiasm. The uniformly enhancing solid portion filled the pituitary fossa, and the cystic component comprised most of the suprasellar portion of the neoplasm.	Transsphenoidal approach: GTR.	N	N	Y, follow-up for 51 months without recurrence.
Ramesh (15)/2013	32/M	Visual dimness; headache; bitemporal hemianopia	No evidence of pituitary dysfunction.	N/A	The lesion located in sellar, with suprasellar extension.	Transsphenoidal approach: The resect extent was not available.	Y, specific radiotherapy method was not available.	N	Y (specific details were not available.)
Belcher (11)/2010	37/M	Sudden occipital headache; bitemporal hemianopia; bilateral optic atrophy; visual acuity decreased; amenorrhea for 6 months; increasing polyuria and polydipsia for 3 months.	Panhypopituitarism; Serum COR 9am: 72nmol/L;	An enhanced mass located at the region of posterior pituitary with suprasellar extension.	N/A	Transfrontal craniotomy: The resect extent was not available.	Y, 4500cGy in 27 fractions over 37 days delivered by a liner accelerator using 3-field technique.	Y	Y. Tumor recurrence 18 years after initial surgery combined radiotherapy, and the patient underwent transfrontal fluid cyst aspiration. Tumor recurred again after another 9 years, with a transsphenoidal surgery was done, but no improvements in symptoms, and another transfrontal resection was done 6-months later. Rradiotherapy or temozolomide therapy is being considered for recurrence currently.
Scheithauer (10)/2009	71/M	Long history of migraine headaches; bitemporal visual field defect, most pronounced in the left superior quadrant.	No evidence of pituitary dysfunction.	2.1×1.8×2.2cm sellar and suprasellar mass.	A 2.1×1.8×2.2cm tumor was homogenous enhancing and to compress the overlying chiasm. It expanded the sella, extended into the left cavernous sinus and suprasellar cistern, eroded the dorsum sellar, and thinned the sellar floor.	Transsphenoidal approach: Subtotal resection.	N	N	Y, follow-up for 2 years, the residual tumor was not changed, and had no effect upon the optic chiasm.
Mukhida (9)/2006	43/M	6-month libido decreasing; loss of weight; myalgias; hot flashes; normal vision and visual field.	TSH:2.25mIU/L (0.35-5.50); FT3: 3.4pmol/L (3.5-6.5); FT4: 6.7pmol/L (11-23); LH: 0.1IU/L (1.5-9.3);FSH :<1.0IU/L (1.4-18.1); PRL:40.7ug/L (3.0-18.0); ACTH:<2.0pmol/L (1.3-15); serum cortsol: 55nmol/L (250-850).	N/A	A sharply circumscribed 1.7 cm mass in the sellar turcica with diffuse homogeneous enhancement of pituitary stalk at the apex of the lesion. The optic chiasm was displaced and slightly compressed by the suprasellar portion of the lesion.	Transsphenoidal approach: GTR, uneventful.	N	N	Y, follow-up MRI for 3-month and 12-month postoperatively without tumor recurrence. Continuing hormone replacement therapy, and the patient felt less lethargic in 3 months.
Thomson (7)/2001	64/M	18-month vision deteriorating; bilateral temporal field loss; panhypopituitarism and taking thyroxine tablets for 1-year.	TSH: 0.07mU/L (0.2-6.0); T: 0.6nmol/L (8.0-27.0); FSH: 0.7IU/L (<8); LH<0.3 IU/L (<8); GH <0.1mU/L; PRL: 817 mU/L (<500)	N/A	A large enhancing mass in the pituitary fossa, and extending into suprasellar cistern; vascular filling defects were seen within the tumor.	Transsphenodial approach: Subtotal resection.	N	N	Y, follow-up 3 months, MRI showed residual tumor, and the patient prepared to undergo radiotherapy.
Chiu (8)/2001	32/M	1-year sensorium disturbing; visual loss; appetite increasing; diabetes insipidus; complete blindness in the right eye, only the lower nasal quadrant in the left visual field was preserved.	Panhypopituitarism. Not given for detailed hormonal changes.	Hyperdense mass in the suprasellar region extended into both the basal ganglia and the left lateral ventricle with intense enhancement. Cystic areas were noted. No calcification.	A lobulated suprasellar mass, with hypo- on T1WI and hyper- on T2WI, and the solid area was intensely enhanced. The sellar was enlarged and eroded, and the 3rd ventricle was elevated and compressed. Cystic areas were present. The basal ganglia were marked distorted.	Transpterional craniotomy. No removal degree given.	N/A	N/A	N/A
Winer (5)/1989	81/M	6-month visual loss; bitemporal hemianopia; complete right third nerve palsy; intermittent double vision; headache; right ptosis; slight postural dizziness.	PRL: 536mU/ml (3-178); Free T4: 7pm/l (>8.8)	A large enhancing mass arising in the pituitary fossa and extending out of sellar.	N/A	Transsphenodial approach: Uneven with no CSF leak. (The resect extent was not available.)	N	N	Y, died of post-operative intracranial infection with a mixture of gram-positive organisms.
Sarkisia (6)/1956	31/F	Secondary amenorrhea; intermittent lactorrhea; 8-month vision worsening (blurred and hazy) for left eye associated with photophobia; bitemporal hemianopsia.	N/A	Without CT, X-ray: Enlarged pituitary fossa with erosion of the posterior clinoid processes.	N/A	Craniotomy: Much of the mass resection.	N/A	N/A	Y, uneventful recovery with good visual improvement of the left eye. No long-term follow-up information available.

**Table 2:** Pathological features of reported cases in the literature.

Author^reference^/ year	Gross specimens	Hematoxylin and eosin staining	Immunohistochemical study	Electron Microscope
Present case	A gray-brown, soft, and friable mass	Well-formed perivascular anucleate zones (pseudorosettes) and (true) ependymal rosettes composed of bland cuboidal or columnar tumor cells.	Ki-67(+3%); EMA(++);S-100(++); Vimentin(++); Syn(-); GFAP(-); CD56(++); Nestin(++); TTF-1(+)	N/A
Liu (13)/2019	Grayish brown, soft, rich in blood supply	Pseudorosette was observed around the blood vessel and ependymal under the microscope, the tumor cells arranged around the blood vessels radially and formed a vascular nonucleated region.	Ki-67(+8%); GFAP(++); EMA(++); P53(+10%)	N/A
Wang (3)/2018	Pinkish, soft, and friable with less blood supply.	Characteristic well-formed ependymomal rosettes and ependymal canals of various sizes, which were scattered throughout the tumor. Ependymomal rosettes were composed of columnar or cuboidal cells arranged around a central tubular lumen, which contained no fiber-rich neuropil or cytoplasmic projections. Perivascular ependymoma rosette was rarely found. The tumor cells were composed of uniform or moderately pleomorphic and somewhat hyperchromatic nuclei with one or two nucleoli, and relatively pale eosinophilic cytoplasm without a clear cytoplasmic border. Nuclear grooves were occasionally seen. Cilia and papillae were noted to be located at the luminal surface.	Ki-67(MIB-1)(<1%); GFAP(+++); S-100(+++); Vimentin(+++); EMA(+); CgA(+); Syn(-), ACTH(-); LH(-); PRL(-); FSH(-); GH(-);	N/A
Lee (12)/2017	Firm and bloody	Highly cellular and composed of compactly arranged small round to oval cells. In area, the tumor showed glandular or papillary structures. These features were very similar to those of pituitary adenoma at a glance. In higher magnification, tumor cells showed somewhat elongated nuclei, and arranged in the fibrillary background, forming perivascular pseudorosettes, papillary configurations, or true ependymal rosettes. Mitoses were not found and there was no necrosis.	Ki-67 (<1%); S-100(+++); Vimentin(+++); GFAP(+); CD99(+);In epithelial membrane antigen staining, characteristic paranuclear dot-like positivity was also noted	N/A
Parish (14)/2015	Grayish orange neoplasm	Relatively low cell density and had a prominent fibrillary background. Rare islands of normalappearing adenohypophyseal cells were encountered. The main component of the mass was made up of broad fascicles of bipolar cells with round to oval nuclei, small clumps of chromatin, occasional small nucleoli, and scanty eosinophilic cytoplasm with long cell processes. In many foci, these cells had a well-defined perivascular pseudorosettes. High mitotic rate, necrosis, or hyperplasia of the vascular endothelium was not observed. Scattered throughout the lesion were many aggregates of hemosiderin-laden macrophages suggestive of previous episodes of hemorrhage.	Ki-67 (<1%); GFAP(+++); staining for pituitary hormones showed no positive cells within the mass;	Ultrastructural studies performed on tissue samples obtained from the paraffin block showed numerous bundles of intermediate filaments and basal corpuscles, cilia, and complex intercellular junctions consistent with the ependymal differentiation of the tumor cells. Postoperatively, the patient did well, but continued to suffer from anterior pituitary insufficiency that required complete hormone replacement.
Ramesh (15)/2013	N/A	Clear ependymal cells with perivascular pseudorosettes and ependymal canal formation, suggestive of clear cell ependymoma	A few cells GFAP(++);	N/A
Belcher (11)/2010	Reddish, firmed mass	Tumor was composed of sheets of cells with crowded but not pleomorphic, pale ovoid nuclei, and no clear cytoplasmic borders. Pseudorosettes were occasionally seen. High grade feature such as necosis, microvascular proliferation or mitoses were not seen. Recurrent tumor showed low-grade ependymoma with prominent pseudrosettes, only occasional mitoses, but no microvascular proliferation or necrosis was seen. Nuclear atypia, most likely a consequence of previous radiotherapy, was noted.	Cytoplasmic ring-like and dot-like positive for EMA and diffuse positivity for GFAP No pituitary or neuronal marker was positive.	N/A
Scheithauer (10)/2009	The tumor appeared gray-white, glistening and relative avascular. It was soft to rubbery, partly firm.	The tumor composed of regular, spindled to plump, cells arranged in sheets and small fascicles. No definite perivascular pseudorosette formation was seen. In areas, ill-defined lobules were somewhat demarcated by collagenous tissue. Nuclei featured open to delicate chromatin and small, central nucleoli. Mitoses were rare, no necrosis was evident. Neither Tosenthal fibers nor granular bodies were seen.	No pituitary hormone staining was detected. GFAP (-); S-100 (++) and EMA (++) ; CAM 5.2 (++); SYN (-); CD34 (-); collagen IV(-); and smooth muscle actin (-). Ki-76 labeling index: 5.3%.	The tumor featured cohesive cells with a moderate nuclear-cytoplasmic ratio, oval to irregular nuclei with variable chromatin and small- to moderate-size nucleoli, as well as occasional processes. Cytoplasm contained little Golgi, scant rough endoplasmic reticulum, moderate numbers of intermediate filaments, rare microtubules, occasional lysosomes. Intra- and intercellular lumens containing microvilli and cilia were noted as were desmosomal junctions. The interface of tumor with stroma featured basal lamina formation. No interdigitation of cell membranes was noted.
Mukhida (9)/2006	N/A	Characteristic nucleus-free zones composed of spindle glial elements with numerous well-formed ependymal rosettes.	GFAP (-); Vimentin (++); EMA (++); S100 (++); adenohypophysial elements (-) (including CK, SYN, chromogranin, LH, FSH, Pit-1 and alpha subuit); neuronal elements (-) (including Neu-N, NF, Neurophysin, Vasopressin); MIB-1 labeling index was less than 1% and p53 was absent	Typical complex, large intercellular junctions as well as large bundles of intermediated filaments.
Thomson (7)/2001	N/A	Monomorphic oval or elongated cells in well-formed perivascular pseudorosettes. An occasional mitotic figure was present, no cytological or architectural atypia.	GFAP (++), especially in tumor cells around blood vessels; negative for pituitary hormones.	N/A
Chiu (8)/2001	N/A	Sheets of polygonal cells with a rich delicate vascular network. The tumor cells possessed round and regular nuclei and pale eosinophilic cytoplasm. Perivascular pseudo-rosettes were scatted throughout the tumor.	GFAP (++), and CK (-)	N/A
Winer (5)/1989	Gray tissue with a gelatinous consistency in some parts.	The tumor was made up of elongated cells arranged in loosely packed rows and perivascular pseudo-rosettes. The tumor cells had eccentric pleomorphic nuclei, coarse chromatin and moderate number of mitotic figures.	GFAP (++) (No other detail information available)	N/A
Sarkisia (6)/1956	The tumor was gray-brown in color, soft, and friable.	Spinal cells with abundant cytoplasm arranged in rosettes and pseudo rosettes and radially around vascular channels.	N/A	N/A

N/A, not available; GFAP, glial fibrillary acidic protein; CK, cytokeratin; SYN, Synaptophysin; NSE, Neuron-specific enolase; GFAP, Glial fibrillary acidic protein; NF, Neurofilament; EMA, Epithelial membrane antigen; CgA, chromogranin A; FSH, follicle stimulating hormone; LH, luteinizing hormone; ACTH, adrenocorticotrophic hormone; PRL, prolactin; GH, growth hormone; +++, strong positive; ++, positive; +, weak positive.

### Molecular Subtypes of Ependymomas

Along with developments in molecular biology techniques, many studies have been carried out on the occurrence and development mechanisms of ependymoma. Pajtler et al. published a DNA methylation profile in 2015, which analyzed the molecular types of ependymomas in different anatomical locations (26), and the classification was adopted by the WHO classification of CNS tumors in 2016. In 2018, Pajtler et al. refined the classification further (27). At present, ependymomas are usually regarded as a molecularly heterogeneous disease entity and are classified into nine distinct molecular subtypes (**Table 3**), according to DNA methylation and gene expression profiling, which have reached a broad consensus (26–28). Supratentorial ependymoma (ST-EPN) includes two molecular subtypes, ST-EPN-RELA (*RELA-C110RF95* fusion type) and ST-EPN-YAP1 (*YAP1* fusing with other genes, with a high frequency of *YAP1-MAMLD1* fusion) (29, 30). ST-EPN-RELA accounts for 72% of ST-EPN and occurs in infants, children, and adults (28). *RELA-C110RF95* fusion protein leads to the abnormal activation of *NF-κB* pathway, leading to a poor prognosis of this subtype (30). Gessi et al. suggested that *p65/RELA* combining with *L1CAM* antibody is also a valuable index to diagnose ST-EPN-RELA (31). Moreover, ependymomas with *RELA* fusion-positive were added to the newly revised WHO classification in 2016 (WHO Grade II-III). ST-EPN-YAP1 occurs primarily in infants and children and usually has an excellent outcome after surgery combined with radiotherapy (26, 27, 30). A European retrospective study showed a 5-year progression-free survival (PFS) rate of <30% and a 5-year overall survival (OS) rate of 75% for ST-EPN-RELA, compared to a 5-year PFS rate of 66% and a 5-year OS rate of 100% for ST-EPN-YAP1 (26, 27). However, these findings need further verification in prospective studies.

**Table 3:** Summary of 9 molecular subtype and clinical characteristics.

Location	Supratentorial (ST-)			Posterior Fossa (PF-)			Spinal (SP-)		
Molecular Subgroup	ST-SE	ST-EPN-YAP1	ST-EPN-RELA	PF-SE	PF-EPN-A	PF-EPN-B	SP-SE	SP-MPE	SP-EPN
Histopathology	Subependymoma	(Anaplastic) Ependymoma	(Anaplastic) Ependymoma	Subependymoma	(Anaplastic) Ependymoma	(Anaplastic) Ependymoma	Subependymoma	Myxopapillary Ependymoma	(Anaplastic) Ependymoma
WHO Grade	I	II/III	II/III	I	II/III	II/III	I	I	II/III
Genetic	Balanced Genome	YAP-1 Fusion	RELA Fusion; Chromothripsis	Balanced Genome	Balanced Genome	Chromosomal Instability	6q Deletion	Chromosomal Instability	NF-2 Mutation
Age Group	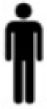	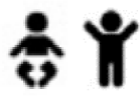	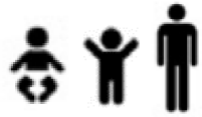	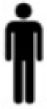	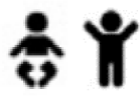	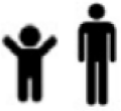	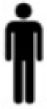	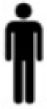	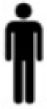
Median Age (y)	40	1.5	8	59	3	30	49	32	40
Outcome	Good	Good	**Poor**	Good	**Poor**	Good	Good	Good	Good
Subgroup Occurrence	4%	3%	18%	7%	48%	10%	1%	5%	4%

Posterior fossa ependymoma (PF-EPN) was classified into two molecular subtypes of PF-EPN-A and PF-EPN-B, according to the methylator phenotype of the promoter CpG island (27). The former exhibits hypermethylation of the CpG island and the latter exhibits hypomethylation. The grouping could also be divided by the global level of lysine 27 on histone 3 (H3K27), with the low epigenetic mark in PF-EPN-A and high in PF-EPN-B (32). PF-EPN-A accounts for 74% of all PF-EPNs and occurs primarily in infants and young children. Moreover, tumors are mainly located in the midline, with high recurrence and metastasis rates, despite gross tumor resection (GTR) (27, 33–35). Chromosome *1q* amplification (15-20% PF-EPN) and the absence of H3K27me3 trimethylation are considered to be associated with a poor prognosis (27, 32, 36, 37). PF-EPN-B occurs primarily in adolescents and adults, with a more favorable prognosis (27, 33). Some PF-EPN-Bs could be cured by GTR alone, and tumor relapse could be controlled by radiotherapy effectively (35). The prognosis is usually favorable for spinal ependymomas, and 43% of cases exhibit *NF-2* mutation (38). However, some recent studies on aggressive spinal ependymomas have characterized them with *MYCN* amplification, anaplastic morphology, and early dissemination along conus medullaris, with usually a poor prognosis (39, 40), necessitating further studies. Vera-Bolanos et al. also reported that spinal myxopapillary ependymomas should be assigned to WHO grade II because the clinical studies do not support a WHO grade I clinical behavior (41).

Besides, some researchers have also reported some other differences in the molecular phenotype. Johnson et al. reported that ST-EPN also exhibits *EPHB2* amplification and *INK4A* absence (42). Some literature suggested that the tumor suppressor gene *RASSF1A* could be expressed in most ependymomas, with high methylation and site-specification (42, 43). The molecular alterations could be discovered with a range of diagnostic tests below. Fluorescence *in situ* hybridization could demonstrate *RELA-C110RF95* fusion and *YAP1* rearrangement of ST-EPN in association with *MYCN* amplification in aggressive spinal ependymomas (30, 44). Immunohistochemistry assessment of the expression of H3K27-trimethylation could be used to distinguish two types of PF-EPN (32).

### Pathogenesis

Due to their rarity, little is known about the underlying causes mediating the development of SEs. One theoretical explanation is that, their occurrence may be a consequence of a neoplastic transformation during the development of either heterotopic ependymal lining cells or embryological remnants of the ependymal lining within the sellar, especially in the infundibular region (6, 7, 10, 13, 14). From an anatomical perspective, in a 10-week (45-mm) human fetus, neurohypophysis develops as an elongated out-pouching of neuroepithelial cells, which encloses a cavity that is continuous with the cavity of the neural tube. At this stage, the infundibulum consists of undifferentiated ependymal cell precursors (3). Particularly, the ependymal cleft, which is not present at birth, is readily identified at 13-weeks during development (60-mm), although it recedes by 16-weeks (112-mm). It is plausible that isolated ependymal cells or embryonic ependymoma remnants are left behind in the infundibulum and neurohypophysis. These cells might undergo subsequent neoplastic transformation (7, 8, 13), giving rise to an ependymoma (3, 5–7, 10). Other hypothesis speculates that SE might be a variant of pituicytoma (10, 22). The hypothesis has been supported by the ultrastructural evidence of pituicyte, a specialized intrinsic cell type of the posterior pituitary gland (3, 45). Pituicytes are classified as major, dark, ependymal, oncocytic, and granular cell types (45). All these are considered different functional forms of a unique cell line with an ependymal phylogenetic origin (21). The ependymal pituicyte remnants might undergo neoplastic transformation (22).

### Tumor Growth Characteristics

Subsequently, we analyzed the tumor growth characteristics of all the reported cases of SEs. First, the tumor mass can be solid [9/13, (69.23%)] or cystic-solid (3/13, [23.08%]) (8, 13, 14), with the cystic component always involving the suprasellar region of the brain (2/3, [66.67%]) (8, 14). Lesions mostly involve both the sellar and suprasellar regions of the brain (12/13, [92.31%], presenting a prominent enlarged sella turcica (11/12, [84.62%]). The pituitary stem appeared consistently raised or displaced (12/13, [92.31%]), and the tumor mass was found to often end up compressing the optic chiasm and optic tract (12/13, [92.31%]). Furthermore, the tumor mass can, in some cases, protrude upward into the third ventricle (2/13, [15.38%]) (8, 13) and affect the basal ganglia regions (1/13, [7.69%]) (8). Ependymoma refers to a ‘plastic’ growth pattern or desmoplastic development that typically protrudes through the outlet foramina into adjacent cisterns (7, 8, 13).

### Clinical Presentation

The mean outset age of SE has been reported to be approximately 46.9 years of age (range: 31-81). In our analysis, we found two female patients and 11 male patients, suggesting a male inclination, consistent with previous reports, suggesting that males have a higher incidence of ependymoma occurrences than females (1, 4). As for the clinical presentation, SEs are often symptomatically similar to nonfunctional pituitary adenomas (18), with some studies reporting that the duration of symptoms also depends on the histological grade of tumors (7). We divided the condition into two aspects here. The first aspect is associated with the mass effect, leading to debilitating symptoms, including headaches, visual deteriorating, bitemporal hemianopsia, and oculomotor paralysis, among others (3). Moreover, cases in which the tumor might grow superiorly can lead to the elevation and compression of the third ventricle of the brain, which might result in hydrocephalus (8). The second aspect is associated with hypopituitarism, leading to weakness, mood swings, hot flashes, weight loss, fatigue, increased appetite, decreased libido, erectile dysfunction, and loss of the body hair. Moreover, patients could have polydipsia and diabetes insipidus if ependymomas involves the posterior pituitary gland (3). However, diabetes insipidus has only been reported in two patients (11, 46). Loh et al. suggested that it can be attributed to the slow growth of SEs (47). In addition, PRL levels could be slightly higher than normal, which is possible due to the so-called “stalk effect” (3), but galactorrhea has been reported only once (6), necessitating further studies. In our patient, PRL levels were found to be significantly raised (1,370.51 mIU/L) compared to normal levels (range: 70.81-566), with no galactorrhea. Furthermore, some reports revealed that the duration of symptoms depends on the histological grade of the tumor (7).

### Radiologic Characteristics

The rarity of SE has led to limited radiographic characteristics, and we have summarized imaging analysis of all 13 clinical cases discussed in this study in **Table 1**. CT scanning frequently shows an enhanced mass arising from the pituitary fossa, extending to the suprasellar region of the brain, and presenting an enlarged sella turcica (3). These ependymomas appear isodense or hyperdense, which can make them difficult to distinguish from pituitary adenoma (3, 7). MRI is regarded as the primary imaging technique for the identification of SEs (3). Although not sufficiently sensitive to diagnose SE on its own, MRI is significant in assessing the location of SEs, demonstrating tumor vascularity, as well as displacement of normal vessels, which are important for surgical planning, especially on sagittal and coronal planes. The lesion is often solid or cystic-solid, with the cystic component usually located in the suprasellar region. Moreover, the tumor solid components appear slightly hypointense on T1WI and slightly hyperintense on T2WI, and the cystic components appear hypointense on T1WI and hyperintense on T2WI (8, 9, 14). Upon enhanced MRI analysis, the tumor margins appear well-defined. In addition, the solid part and cyst wall observed in these tumors are frequently enhanced. Intermixing of poorly enhanced areas is frequently shown in solid parts, suggesting a vascular filling defect (7, 48), not usually seen in pituitary adenomas (1, 7). In some studies, it was suggested that the vascular filling defects observed on MRI scans might help distinguish pituitary adenomas from ependymomas (7). Furthermore, the cystic area of a lesion may follow fluid signal intensity on both T2WI and on the fluid-attenuated inversion recovery (FLAIR) sequence or might remain hyperintense on FLAIR due to the presence of proteinaceous fluid contents (7). Lukashova-v Zangen et al. (49) demonstrated that the isointense signal is shown on the diffusion-weighted imaging. Another characteristic of SEs is the flow-void phenomenon, which can be seen on the plain view of the MRI (13).

### Pathology Features

We summarized the pathological examination of all 13 cases, including our case in **Table 2**. SEs are characterized by various-sized and well-formed true ependymoma rosettes and/or perivascular pseudorosettes scattered throughout the tumor as seen under H&E staining examination (3, 9, 10, 12, 13). Using light microscopy, these rosettes provide strong evidence of ependymal differentiation. The histological features of these rosettes were observed in all 13 cases studied (13/13 [100.0%]), with 5 cases (5/13 [38.46%]) presenting both pseudorosettes and true ependymoma rosettes, 6 cases (6/13, [46.15%]) presenting pseudorosettes, 1 case (9)(1/13 [7.69%]) presenting true ependymoma rosettes only, and 1 other case (10) (1/13 [7.69%]) presenting neither ependymoma rosettes nor pseudorosettes. Perivascular pseudorosettes seem to be more sensitive for the diagnosis of ependymomas than true ependymal rosettes (2, 50). On the other hand, perivascular pseudorosettes are less specific in that they can also be found in medulloblastomas, glioblastomas, and central neurocytoma malignancies (50). Importantly, with the exception of rosette-like structures, the nuclear groove is also a specific cytologic finding for the diagnosis of SEs, which has not been detected in other CNS tumors (3, 51). Kumar et al. (51) compared the cytologic findings of 21 cases of classic ependymomas with other CNS tumors and found ependymal rosettes (100%), nuclear grooves (71.4%), perivascular pseudorosettes (52.4%), and acinar structures (33.3%) throughout these cases.

Immunostaining analysis provides additional significant information for the correct diagnosis of ependymomas, with positive expression levels of TTF-1, GFAP, S-100, vimentin, EMA, and negative expression levels for secreted pituitary hormones and neural markers, such as synaptophysin (10–12, 18, 21). In some cases, GFAP did not appear to be immunoreactive (52), consistent with 3 out of 13 reported cases of SE, including our patient. Moreover, expression levels of the low molecular weight protein and keratin could be observed in approximately 20% of ependymomas (9). Another notable consideration is that the diagnosis of SEs cannot always be made solely based on histological examinations and immunohistochemical analyses. As in the case reported by Scheithauer et al. (10 *)*, histological examination and immunohistochemical analysis of the patient were not straightforward. In difficult-to-diagnose cases, ultrastructural analysis by electron microscopy is imperative.

We found that electron microscopy examination was carried out in 3 cases (3/13, [23.08%]) of SE (9, 10, 14). Here, we have reviewed previous reports of SEs and summarized important ultrastructural features. Cilia and microvilli structures located at the luminal surface of these neoplastic ependymal cells, as well as intercellular junctional complexes located on their lateral surfaces, can be usually observed using an electron microscope (9, 10, 14, 45). In addition, the cytoplasm of these tumor cells contains a moderate to a large number of intermediated filaments and few Golgi, rough endoplasmic reticulum, or microtubules (10, 14, 45). A basal lamina formation can also be present at the interface between tumor cells and vascularized stroma (10, 14), whereas neurosecretory granules have been noticeably absent (3).

The different diagnoses obtained are quite extensive, including pituitary adenoma, craniopharyngioma, chordomas, Rathke’s cleft cyst, meningioma, glioma, and aneurysms, among others (8, 10, 14, 53). Here, we analyzed the preoperative diagnosis of the 13 cases evaluated, of which, 9 cases (9/13 [69.23%]) of pituitary adenomas, 4 cases (4/9 [44.44%]) of pituitary macroadenoma among them, 1 case (1/13 [7.69%]) of glioma, and 1 case (1/13 [7.69%]) of craniopharyngioma, were identified.

### Treatment

#### Surgery

Despite advances in many other tumor types in past decades, maximal safe tumor resection followed by postoperative adjuvant radiotherapy remains the most viable treatment modality for ependymomas (21, 24, 54, 55). Currently, there is no established treatment protocol for SE patients. Surgical resection is still the primary treatment choice with the aim of GTR, as the extent of resection is one of the most important predictors of recurrence and survival (3, 11, 12). Surgical paradigms include the transsphenoidal and craniotomy approach, and the choice depends on the tumor location and its suprasellar extension. All of the 13 clinical cases studied here (including our patient) underwent surgical resection, 8 cases (8/13 [61.54%]) using the transsphenoidal approach and 5 cases (5/13 [38.46%]) using the craniotomy approach, respectively (**Table 1**). When using the transsphenoidal approach, three cases (3/8 [37.5%]) underwent GTR, two cases had a subtotal resection (2/8 [25.0%]), and one case had a partial resection (1/8 [12.5%]). Notably, for two of these cases (2/8 [25.0%]), the extent of resection was not reported. Using the craniotomy approach, one case underwent GTR (1/5 [20.0%]), two cases (2/8 [40.0%]) underwent subtotal resection, and for two cases (2/8 [40.0%]), the extent of the resection was not reported. Our patient underwent a transfrontal-temporal approach of total tumor resection. Compared with incomplete resection, local recurrence and metastatic seeding significantly reduced, and the 5- and 10-year survival rates were prolonged after GTR (16, 56). Relapse of ependymomas is usually treated with surgery or re-radiotherapy, and no chemotherapeutics or targeted agents have shown significant survival benefits to date (57, 58).

#### Radiotherapy

The use of radiotherapy modalities to treat ependymoma remains controversial, especially in children (59). Whipple et al. reported that radiotherapy is imperative for residual tumors (18); however, no objective data currently supports this theory (3). As for SE, 4 cases (4/13 [30.77%]) underwent postoperative radiotherapy, and only one case (1/13 [7.69%]) showed tumor recurrence (11). Due to the limited number of patients reported, we do not know whether postoperative radiotherapy is a feasible and effective therapy for patients with SE. In some reports, it was indicated that postoperative local radiotherapy could be considered if the residual tumor was distant from the optic apparatus based on postoperative MRI scan analysis (11, 12), and this might be feasible. The patient reported here did not accept radiotherapy as the postoperative CT and follow-up MRI showed complete tumor resection and no tumor recurrence sign.

#### Chemotherapy

Chemotherapy has not been a routine treatment paradigm for ependymomas, and no patient with SE has been reported to undergo chemotherapy (2). However, chemotherapy is advised for some ependymomas following relapse, particularly when the patient has already undergone radiotherapy or as an attempt to defer radiotherapy and associated adverse effects, including stroke, neurocognitive deficits, and secondary malignancy, among others (59). Generally, chemotherapy for SE remains to be studied, although it might have some benefits as salvage treatment when there are no other options (2, 11). We do not advocate immediate postoperative chemotherapy. However, when dealing with recurrent disease and/or complex SE, according to a report by Belcher et al., the chemotherapeutic agent temozolomide could be considered a treatment option (11).

#### Molecular Targeted Therapy

Drugs targeting mutant fusion genes are not available and are under investigation (21, 24, 54, 55). The roles of NF-κB inhibitor and YAP-1 inhibitor have been verified in the pre-clinical studies in animal models (60). The hypermethylation of CpG island and aberrant histone modification were confirmed as a reversible chemical modification. Deepening of basic research will verify whether they are expected to be an effective, potential drug target. Moreover, DNA methyltransferase inhibitors (5-Azacytidine and decitabine) and histone deacetylase inhibitors (HDACIs, suberoylanilide hydroxamic acid [SAHA], and romidepsin) are currently used in clinical practice, mainly to treat hematological malignancies (61, 62). Mack et al. also verified that decitabine, SAHA, and GSK343 (the competitive inhibitor of HDACI) could inhibit the growth of ependymoma cells during *in vitro* and *in vivo* tests (34).

### Prognosis

Patients with ST-EPN-RELA and PF-EPN-A comprise the largest molecular subtypes and usually have a poor prognosis (63). The prognosis of SEs is difficult to assess given the small number of reported cases in the medical literature, and no study is available on the molecular subtypes of SE. Nevertheless, as shown in **Table 1**, the overall reported outcome for these patients is promising. The prognosis of ependymoma is associated with the age at onset, tumor location, the extent of initial surgical resection, and histological tumor grade (2, 4, 11). The only factor that a care provider can truly influence is the extent of tumor resection; therefore, maximum tumor resection should be the final surgical aim. Another consideration worth noting is that the recurrence of ependymomas is usually relatively late (23). Belcher et al. reported a patient of SE, who exhibited recurrence twice, and the first and second recurrence cases occurred 18 and 27 years after the initial surgery, respectively (11). Moreover, distant and cerebrospinal metastases appear to be associated with an incomplete initial excision and high-grade tumor histology (11). In summary, maximum tumor resection combined with radiotherapy and close follow-up using MRI analysis is highly encouraged in these patients.

### Limitations

The main limitation of this study is a lack of genetic analysis and molecular subtyping.

## Conclusion

In this study, we reported a patient with SE, which is exceedingly rare, with only 12 cases having been reported to date. GTR usually provides the best chance for long-term survival. However, the pathogenesis and recurrence rate remain unknown. Besides, the therapeutic modality has not been standardized, requiring further validation. Thus, more reported cases and long-term follow-up studies are necessary to increase knowledge about this disease. Moreover, the classification of ependymoma that combines molecular phenotype and clinical characteristics will help determine the prognosis and risk classification of patients more accurately, providing an important basis for individualized treatment in the future. The establishment of different molecular subtypes of ependymoma cells and animal models is the key to screening new treatment modalities.

## Data Availability Statement

The original contributions presented in the study are included in the article/supplementary material. Further inquiries can be directed to the corresponding author.

## Ethics Statement

The studies involving human participants were reviewed and approved by the ethics committee of the First Hospital of Jilin University. The patients/participants provided their written informed consent to participate in this study. Written informed consent was obtained from the individual(s) for the publication of any potentially identifiable images or data included in this article.

## Author Contributions

LZ and YJ made study design, data collection, data analysis and interpretation, and composed the manuscript and literature review. YL and YW were the surgeon that performed the surgery and did data collection, data analysis, and interpretation. LL and YB made English and grammar corrections, critical revisions, and approved final version. LZ made great effort in the modification of the article. LZ and YL had the acquisition, analysis or interpretation of data for the work, revising it critically for important intellectual content, final approval of the version to be published, and agreement to be accountable for all aspects of the work in ensuring that questions related to the accuracy or integrity of any part of the work are appropriately investigated and resolved. All authors contributed to the article and approved the submitted version.

## Conflict of Interest

The authors declare that the research was conducted in the absence of any commercial or financial relationships that could be construed as a potential conflict of interest.

## Abbreviations

CNS, central nervous system; CT, computerized tomography; EMA, epithelial membrane antigen; FLAIR, fluid attenuated inversion recovery; GFAP, glial fibrillary acidic protein; GTR, gross tumor resection; H&E, hematoxylin and eosin; H3K27, lysine 27 on histone 3; HDACI, histone deacetylase inhibitor; OS, overall survival; PF-EPN, posterior fossa ependymoma; PFS, progression-free survival; PRL, prolactin; MRI, magnetic resonance imaging; SAHA, suberoylanilide hydroxamic acid; SE, sellar ependymoma; ST-EPN, Supratentorial ependymoma; T1WI, T1-weighted imaging; T2WI, T2-weighted imaging; TTF-1, thyroid transcription factor-1; WHO, World Health Organization.
