# Erratum: Case Report: Sellar Ependymomas: A Clinic-Pathological Study and Literature Review

**DOI:** 10.3389/fendo.2021.748952

**Published:** 2021-09-02

**Authors:** Frontiers Editorial Office

**Affiliations:** Frontiers Media SA, Lausanne, Switzerland

**Keywords:** diagnosis, ependymoma, molecular subtype, pituitary tumor, sellar ependymoma, treatment

Due to a production error, the first three authors were listed as co-first authors. The correct author list appears below.

The publisher apologizes for this mistake.

Liyan Zhao^1^, Yining Jiang^2^, Yubo Wang^2^, Yang Bai^2^, Liping Liu^1^ and Yunqian Li^2*^

The original version of this article has been updated.

